# Recurrence and Familial Inheritance of Intronic *NIPBL* Pathogenic Variant Associated With Mild CdLS

**DOI:** 10.3389/fneur.2018.00967

**Published:** 2018-11-27

**Authors:** Maura Masciadri, Anna Ficcadenti, Donatella Milani, Francesca Cogliati, Maria Teresa Divizia, Lidia Larizza, Silvia Russo

**Affiliations:** ^1^Laboratorio di Ricerche di Citogenetica Medica e Genetica Molecolare, Istituto Auxologico Italiano (IRCCS) Milan, Italy; ^2^Centro Regionale Malattie Rare, Azienda Ospedaliero Universitaria Ospedali Riuniti Ancona, Italy; ^3^Pediatric Highly Intensive Care Unit, Department of Pathophysiology and Transplantation, Fondazione IRCCS Ca' Granda Ospedale Maggiore Policlinico (IRCCS) Milan, Italy; ^4^UOC Genetica Medica, Istituto Giannina Gaslini (IRCCS) Genoa, Italy

**Keywords:** Cornelia de Lange, familial inheritance, recurrent variant, intronic variant, mild phenotype

## Abstract

Splicing pathogenic variants account for a notable fraction of *NIPBL* alterations underlying Cornelia de Lange syndrome but are likely underrepresented, due to overlooking of non-canonical intronic variants by traditional and contemporary sequencing methods. We describe five subjects, belonging to three families, displaying a mild Cornelia de Lange syndrome phenotype who carry the *NIPBL* pathogenic variant c.5329–15A>G, affecting the IVS27 branch site, yet reported in a single case. By RNA analysis we evidenced two alternative transcripts: the exon 28 in frame skipped transcript, described in the published case and an out-of-frame transcript retaining 14 nucleotides of IVS27 3′end. Even if both aberrant transcripts are at negligible levels, their presence justifies the CdLS phenotype shared by our patients consisting of borderline-mild cognitive impairment and slight but typical facial dysmorphisms. Transmission of the pathogenic variant from pauci-symptomatic mother to her siblings emphasizes the need of molecular diagnosis extended to deep intronic regions in patients with subtle but recognizable CdLS phenotype.

## Introduction

Cornelia de Lange Syndrome (CdLS1 MIM 122470) is a developmental multiple congenital anomalies disorder with intellectual disability characterized by distinctive facial features as synophrys with highly arched eyebrows, long eyelashes, short nose with anteverted nares, small widely spaced teeth, microcephaly, pre- and post-natal growth retardation, hirsutism and subtle or evident upper limb defects (1). Prevalence estimates range from 1:100,000 to 1:10,000 (2, 3) though they precede the finding of the surprisingly high fraction of individuals with somatic mosaicism (4) and the application of high resolution diagnostic tools, such as deep sequencing on multigene panel/exome or transcript analysis, which all together have enhanced the detection of pathogenic variants. Up to 70% of clinically diagnosed CdLS patients harbor heterozygous pathogenic variants in *NIPBL* gene (OMIM 608667) (5, 6) which encodes a regulatory protein loading the cohesin complex onto sister chromatids. More than 300 pathogenic variants, mostly point mutations, have been found evenly spread across the whole *NIPBL* gene in CdLS patients (4, 7). Frameshift variants, the most prevalent *NIPBL* defects, commonly underlie a severe phenotype, while pathogenic missense variants are often associated to mild disease (8). Splicing alterations account for 17% *NIPBL* pathogenic variants (9) but are likely underestimated, as almost all those detected affect canonical splice sites or flanking consensus sequences (LOVD *NIPBL*). A familial case where a canonical *NIPBL* splice variant segregating from a mosaic mother to her son was recently reported to be associated to heterogeneous phenotype expression (10). The prevalence of pauci-symptomatic *NIPBL-*mutated cases is difficult to estimate as these patients are often overlooked and do not receive molecular diagnosis. A mild CdLS phenotype that retains many characteristic facial features but presents with low grade intellectual disability and minor malformations has been shown to be underpinned by non-canonical splicing variants by Teresa-Rodrigo (11) who described two intronic *NIPBL* pathogenic variants in two mild CdLS German patients. We herein report on five Italian patients with mild CdLS phenotype, three of whom belonging to the same family, who carry the same branch site substitution detected in one of the two above mentioned mild CdLS patients (11). This data corroborates the contribution of non-canonical splicing variants to *NIPBL*-caused CdLS and enhances optimization of detection rate in slightly affected subjects who are potential transmitters of the disease.

## Case report

All five patients from three unrelated families were referred to our lab by experienced clinical geneticists with a diagnostic query of mild CdLS phenotype.

### Family 1

**Figure 1:**
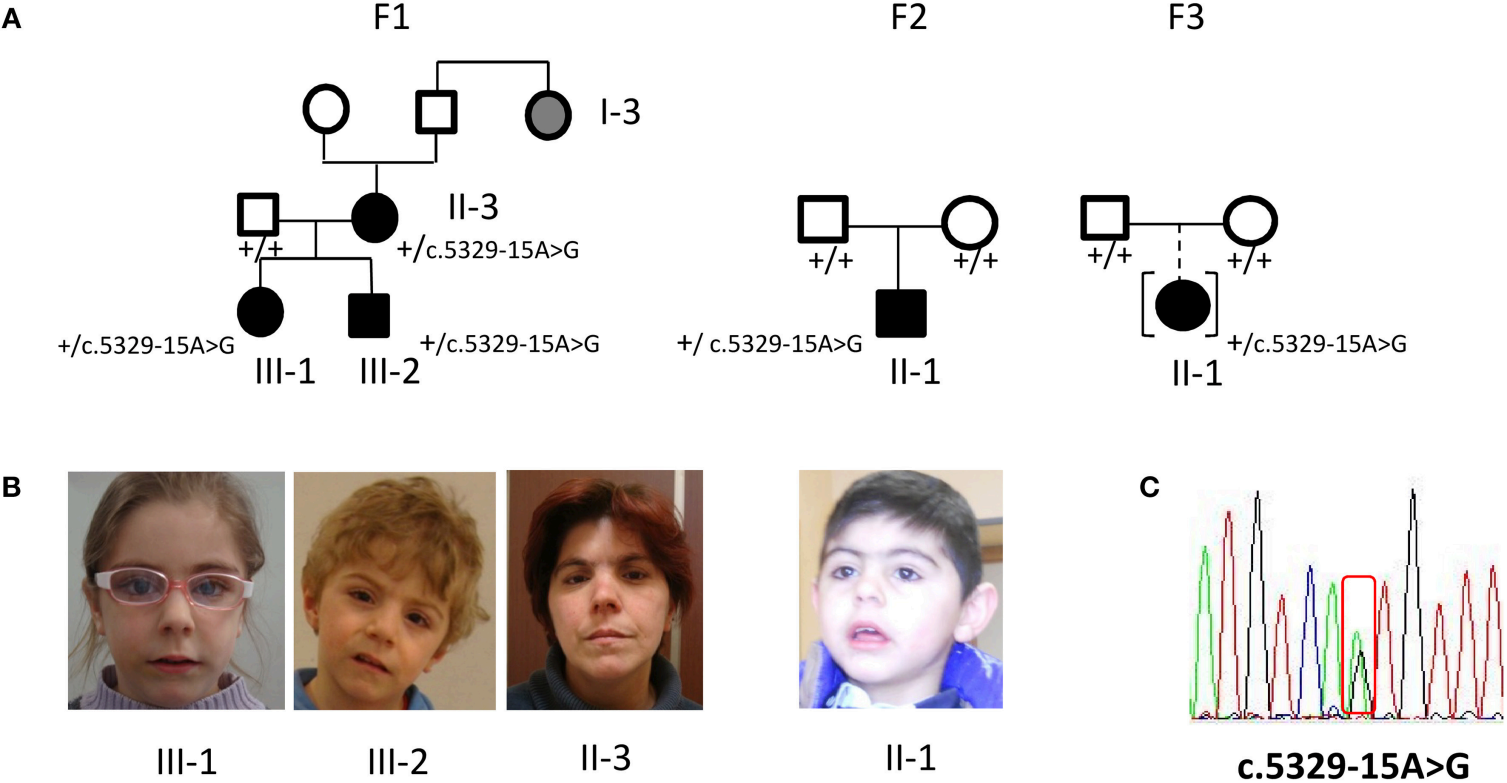
Phenotype and molecular findings of five CdLS patients with the *NIPBL* intronic variant c.5329-15A>G. **(A)** Pedigrees of family 1 (F1) with three ascertained affected individuals (II-3, III-1, III-2) and one inferred patient and of family 2 (F2) and 3 (F3) sporadic cases. Black symbols are for the CdLS patients, gray symbol for individual with inferred phenotype; black symbol in square brackets indicates an adopted child. **(B)** Facial appearance of F1 sibs and mother and of F2 proband. **(C)** DNA pathogenic variant (cDNA nomenclature). Below each family member the normal (”+”) or variant allele (c.5329-15A>G) is indicated.

Family 1 comprises a female and a male (III-1, III-2) siblings referred at the age of 4 and 2 years, respectively, and their 37 years- old mother (II-3). All subjects displayed mild, but typical signs of CdLS facial gestalt as synophrys, long filtrum, and anterved nostrils (Figures 1A,B). Family history recorded similar facial features for II-3's paternal aunt (I-3). The clinical diagnosis was supported by neuropsychological assessment of the siblings, evidencing mild to moderate intellectual disability (ID).

### Family 2

Proband II-1 is a male child, born to healthy parents (Figure 1A) referred at the age of 5 years because of facial gestalt typical of CdLS (Figure 1B) and mild psychomotor delay. He was delivered preterm (36+6 weeks of gestational age) by C-section. The neonatal parameters were: length 45 cm, weight 1.840 g, head circumference 29 cm, APGAR score 7/8. He was transferred in NICU for respiratory distress, tachipnoea, and hypoglycaemia.

Postnatally, he demonstrated mild growth restriction for all parameters (head circumference, height and weight) and a mild truncal hypertonia. At the age of 6 months, a lacrimal duct stenosis was diagnosed. At the age of 5 years, weight was 13 kg, height 98.2 cm, head circumference 45 cm, and psychomotor development was mildly delayed. He displayed diffuse hirsutism.

### Family 3

The proband II-1 is an adopted girl (Figure 1A) referred at age of 10 years for scholar problems and suggestive facial dysmorphisms. Whether she is a sporadic or a familial case cannot thereby be assessed. As shown in Figure 1A, CdLS facial gestalt was characterized by synophrys, highly arched eyebrows, short nose with anteverted nares, long philtrum, thin vermilion of the upper lip, high, and arched palate. Table 1 reports for all five patients growth parameters referred to the standard growth charts, at birth and at last clinical evaluation, as well as, feeding problems, developmental milestones and cognitive and behavior impairment. All the patients displayed pre- and post-natal growth delay, craniofacial dysmorphisms and mild psychomotor delay, which raised the clinical suspicion of mild CdLS phenotype. Conversely, other recurrent CdLS signs were absent or underrepresented including gastroesophageal reflux (GER), not observed in any of the patients, hirsutism displayed only by the proband of family 2, and subtle limb anomalies present in the two sporadic cases. As regards intellectual disability, three unrelated patients (III-2 from F1, and the probands of F2, and F3) are ranked borderline, while the mother of family 1 is assessed at the lowest level of normal range for IQ. Slight intra-familial variability is attested by the moderate ID of III-1 from F1; both sibs display behavior anomalies, III-1 is aggressive, hyperactive and more recently hyperphagic, while her brother III-2 shows somnolence, asthenia, and hyperactivity at school.

**Table 1:** Clinical features of five CdLS individuals with NM_133433.3: c.5329-15A>G *NIPBL* pathogenic variant.

	**Family 1**			**Family 2**	**Family 3**
**Probands**	**III-1**	**III-2**	**II-3**	**II-1**	**II-1**
Clinical severity	Moderate	Mild	Mild	Mild	Mild
Gender	F	M	F	M	F
**ANTHROPOMETRIC DATA (BIRTH)**					
Gestational age	40	39	40	36+6	39
Weight (Kg)	10 cen	10 cen	< 3 cen	< 3 cen	< 3 cen
Length (cm)	10 cen	na	na	3 cen	25 cen
OFC (cm)	< 3 cen	na	na	< 3 cen	< 3 cen
IUGR	+	+	–	+	+
**ANTHROPOMETRIC DATA**					
Age at last evaluation	4 years	2 years 3 months	37 years	7.5 years	10 years
Weight (Kg)	10 cen	< 3 cen	75–90 cen	< 3 cen	25 cen
Length (cm)	25 cen	< 3 cen	10 cen	< 3 cen	10 cen
OFC (cm)	na	< 3 cen	na	< 3 cen	10–25 cen
Postnatal growth retardation	+	+	+	+	nd
Feeding difficulties/swallowing disorders	+	–	Na	+	+
GERD	–	–	–	na	–
Hirsutism	–	–	–	++	–
Limb defects	Hip dysplasia	center leg la^a^	–	Proximal thumb set	Partial cutaneous 2°-3° toes syndactyly/clinodactyly 4°-5° toes
**DYSMORPHISMS**					
Synophrys	+	+	+	+	+
Long philtrum	+	+	+	+	+
Anteverted nostrils	+	+/–	+	+	+
Thin upper lip	+	+	–	–	+
Microretrognathia	+	–	–	–	–
**DEVELOPMENTAL DELAY**					
Age of walking (months)	16	19	na	15	19
First words (months)	13–14	23	na	18	22
Microcephaly	+	+	+/–	na	na
Intellectual disability	+	+/–^b^	–	+/–	+/–^c^
Behaviour anomalies	++	+	+	–	+/– (attention deficit disorder)
Others	Mild hypoplasic aortic arch	Scoliosis	na	Lacrimal ducts stenosis	Recurrent infections

a *Lateralized Asymmetry*.

b *70 by Griffith evaluation (borderline range 70–79)*.

c *75 by WISC-IV (borderline range 70–79)*.

## Materials and methods

### Genomic sequencing

Genomic DNA was extracted from peripheral blood leukocytes using Freedom Evo TECAN extractor. A panel of CdLS genes including *NIPBL* (NM_133433), *SMC1A* (NM_300040), *SMC3* (NM_606062), *HDAC8* (NM_300269), and *RAD21* (NM_606462) was interrogated. Genomic sequencing of whole coding region including 20 nucleotides of flanking intron-exon junctions was performed by Illumina Nextera Rapid Capture Enrichment protocol, following the manifacturer's instructions, while the uncovered genomic regions were analyzed by Nextera-XT-Library-prep protocol (Illumina) and Sanger sequencing using the Big Dye Terminator v.3.1 Cycle Sequencing Kit (Applied Biosystems). Intronic *NIPBL* nucleotide variant NM_133433:c.5329-15A > G was confirmed by PCR with primers for genomic DNA 5′-CACCTGTACTGATTTTTAAGTTAAACTTTGA-3′ (forward strand) and 5′-TTTTCACCAAGAACTCGTAGGATCT-3′ (reverse strand) and Go Taq Hot Start Polymerase (Promega).

### cDNA analysis

Total RNA was extracted from peripheral blood leukocytes using Tempus Spin RNA Isolation Kit (Applied Biosystems) and reverse transcribed to cDNA by SuperScript VILO Kit (Invitrogen). cDNA was amplified by Go Taq Hot Start Polymerase (Promega) and sequenced using the Big Dye Terminator v.3.1 Cycle Sequencing Kit (Applied Biosystems) with primers 5′-TATAGCCCAGTGGTTTCGAG-3′ (forward strand) and 5′-GGTCGACAAAGGACAAATCG-3′ (reverse strand) and run on ABI PRISM 3500 sequencer (Applied Biosystems) according to the manufacture's protocol. Electropherograms were compared to *NIPBL* NM_133433 reference sequence.

### Molecular characterization

All the five subjects with a suspected mild CdLS clinical diagnosis were found to carry the *NIPBL* non-canonical intronic sequence alteration NC_000005.9 g.37022138 A>G NM_133433.3:c.5329-15A>G (Figure 1C), characterized only in one German CdLS patient with mild phenotype (11). Whole *NIPBL* gene coding sequence and intronic flanking regions were sequenced, excluding the occurrence of VUS (variant unknown sequence) or pathogenic variants. No pathogenic variants were disclosed neither in the HDAC8, SMC1, SMC3, and RAD21 gene.

**Figure 2:**
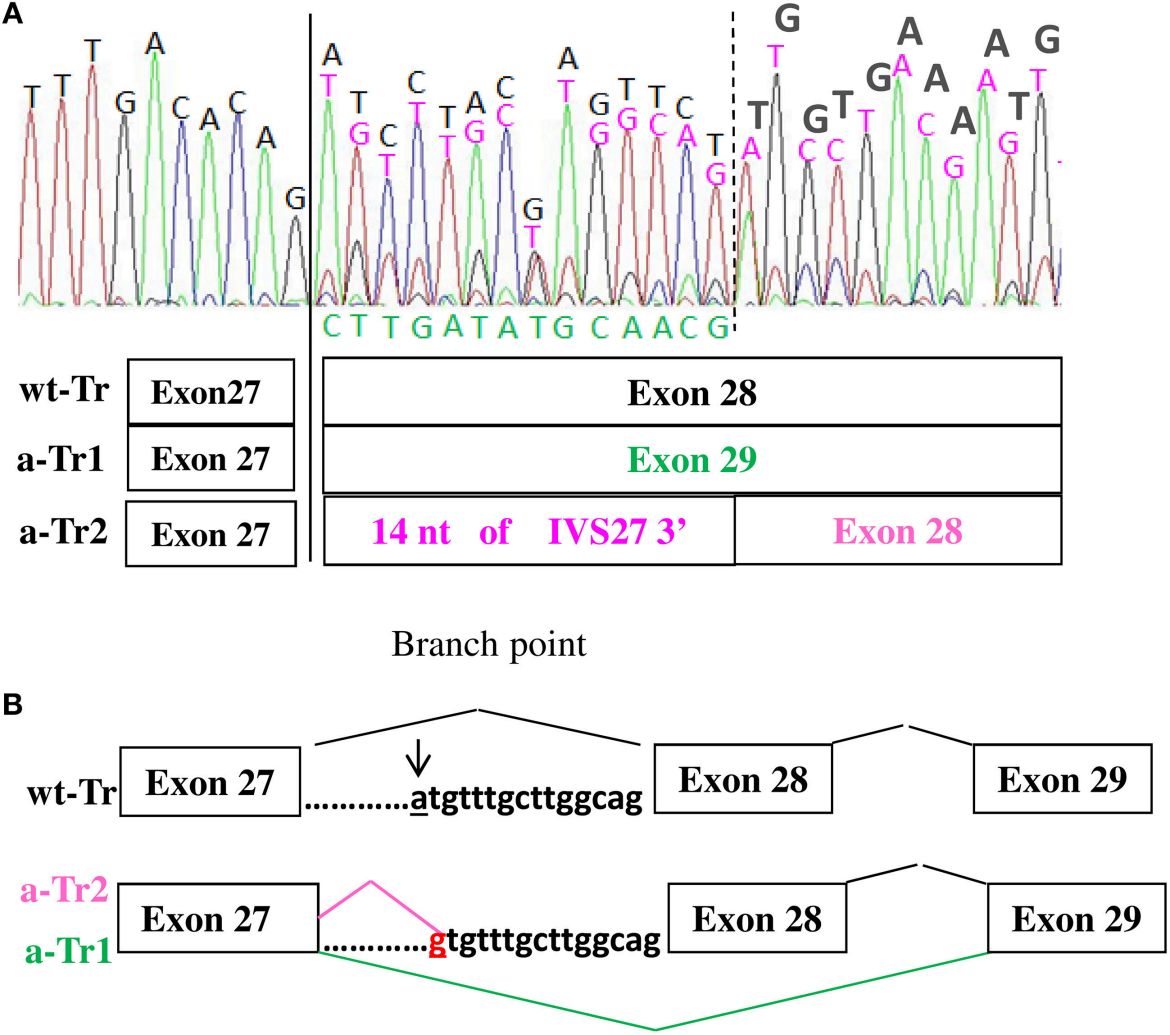
**(A,B)** cDNA electropherograms show the wild type transcript (black characters) and two aberrant transcripts, the in frame a-Tr1, skipping exon 28 (green characters) and a-Tr2 retaining 14 nucleotides of IVS28 3' (purple characters). The intronic “g” replacing “a” hence creating an alternative donor splice site is in red.

Characterization at transcript level of all five patients using both *ad hoc* designed primers and the primers used by Teresa-Rodrigo et al. evidenced besides the expected wild type (wt) transcript, two aberrant transcripts (a-Tr) which sequences are provided in Figure 2A. Namely a-Tr1 is the in frame exon 28 skipped transcript (diagrammed in Figure 2B) found in the German patient, leading to the in frame deletion p (Ile1777Arg1809del). We also detected an additional aberrant transcript (a-Tr2), which sequence retains 14 nucleotides of the 3′ terminus of IVS27, downstream the intronic A>G transition (Figure 2A), leading to frame disruption. The c.5329-15A>G transition affects the recognition of the branch point and the acceptor splice site of intron 27, hence activating a cryptic splice site (diagrammed in Figure 2B). The intronic variant changes a-Tr2 coding sequence into (NM_133433):r.5328_5329ins[5329-14_5329-1;uguuugcuuggcag], predicting the truncated protein p.(Ile1777CysfsX22).

The intronic variant has not been found in the consulted GnomAD, ExAC, and Ensemble databases. According to the ACMG criteria the variant herein described may be considered at strong evidence of pathogenicity PS4 (12), because of its prevalence in CdLS individuals, namely 6 cases including the previously published one (11) and absence in the control population's databases. The cosegregation with the CdLS phenotype within family also features as supporting evidence, PP1 (13).

According to the cDNA electropherogram in Figure 2A, showing the mix of wild type and alternative transcripts, the wild type sequence is prominent.

## Discussion

We have identified in our CdLS patients cohort five individuals with mild phenotype sharing the same c.5329-15A>G *NIPBL* intronic variant, which has been reported to date only in one patient (11). Our findings on three unrelated patients in our cohort of 110 *NIPBL*-mutated cases (2.7%) highlight a mutation hotspot in a non-canonical site of the consensus splice sites (12). The frequency of deep intronic variants affecting the branch site may be underestimated because both Sanger sequencing and NGS not always disclose sequence changes disrupting splicing. Interestingly, three out of the five investigated Italian CdLS patients belong to the same family (F1) (Figure 1A) underlining the yet unreported inheritance of this pathogenic variant and the need of appropriate genetic counseling to families. To date only about a dozen branch site pathogenic variants have been reported (14).

Although the estimated frequency of *NIPBL* splicing defects is consistent (9, 10), only two non-canonical splice variants are recorded in HGMD database: NM_133433:c.459-9G>A which effect is only predicted (4) and the branch site variant NM_133433:c.5329-15A>G, recurring in our patients and the previously described case (11). Sequencing of the proband's cDNA showed two aberrant transcripts a-Tr1 and a-Tr2, a finding consistent with the frequent detection of multiple mRNA species, widely reported for variants deeply located within an intron as those affecting the branch point (15).

According to Teresa Rodrigo, the level of total *NIPBL* expression was not significantly decreased in the characterized case, suggesting negligible production of the in frame aTr-1, likely due to minor use of the alternative acceptor splice site. As regards the out frame a-Tr2, found in our cases, it appears remarkably less represented than the wild type transcript, but more represented than a-Tr1 (see Figure 2A). Interestingly despite only a tiny fraction of the total *NIPBL* mRNA seems represented by the alternative transcripts, likely undergoing degradation, their occurrence is sufficient for recognition of the CdLS phenotype. In conclusion, our data corroborates the previously reported case with the same branch site pathogenic variant (11) highlighting the significance and diagnostic impact of non-canonical intronic variants of *NIPBL* gene in CdLS patients and the graded clinical presentation of CdLS with detection of extremely mild adult cases through their progeny (10). It is well-known that the approximate detection rate of *NIPBL* pathogenic variants (~70%) also implemented by the recent widespread application of multigene panel and WES NGS (9), underestimates the fraction of cases with mild phenotype. Our study first reports on a family segregating through two generations an intronic *NIPBL* pathogenic variant and raises the attention on provision of genetic counseling also when mild dysmorphic features and borderline intellectual disability are noted by the clinical geneticist. Mildly affected CdLS subjects are rare (1) and often they remain molecularly unsolved. Whenever they display suggestive clinical features they should be accurately investigated, also in the non-coding sequence, considering the 50% risk of generating affected progeny (10, 16).

## Concluding remarks

Two main conclusions may be drawn by the present work.

The first one points to the relevance of deep intronic sequence variations, such as those affecting the branch site as disease causing. Sequencing of non-coding regions should be addressed when the patient's phenotype is suggestive of a specific genetic diagnosis. Branch site variants have been rarely described and every further example is instrumental to learn on the multiple consequences of mis-splicing. The second conclusion, inferred by the reported inheritance of Cornelia de Lange resulting from pathogenic variants leading to mis-splicing, underlines the need of detecting the molecular basis of recognizable mild clinical phenotypes to provide genetic counseling to the families.

## Ethics statement

This work was conducted in the context of a research aimed at enhancing molecular diagnosis of CdLS. Patients' parents provided written informed consent to the genetic test and authorized to publish the study including the photos.

## Author contributions

MM and SR conceived and performed the experiments. AF was in charge of management and follow-up of family 1, DM of family 2, and MD of family 3. MM, FC, LL, and SR drafted the manuscript. LL and SR revised the manuscript approved by all the Authors.

### Conflict of interest statement

The authors declare that the research was conducted in the absence of any commercial or financial relationships that could be construed as a potential conflict of interest.

## References

[1] Deardorff MA Noon SE Krantz ID Cornelia de Lange Syndrome. In: Pagon RA Adam MP Ardinger HH Wallace SE Amemiya A Bean LJH editors. GeneReviews®. Seattle, WA: University of Washington (2005). 20301283

[2] Pearce PM Pitt DB Six cases of de Lange's syndrome; parental consanguinity in two. Med J Aust. (1967) 11:502–6. 6022911

[3] Opitz JM. The Brachmann-de Lange syndrome. Am J Med Genet. (1985) 22:89–102. 10.1002/ajmg.1320220110 3901753

[4] Huisman SA Redeker EJ Maas SM Mannens MM Hennekam RC. High rate of mosaicism in individuals with Cornelia de Lange syndrome. J Med Genet. (2013) 50:339–44. 10.1136/jmedgenet-2012-101477 23505322

[5] Tonkin ET Smith M Eichhorn P Jones S Imamwerdi B Lindsay S. A giant novel gene undergoing extensive alternative splicing is severed by a Cornelia de Lange-associated translocation breakpoint at 3q26.3. Hum Genet. (2004) 115:139–48. 10.1007/s00439-004-1134-6 15168106 PMC4894837

[6] Krantz ID McCallum J DeScipio C Kaur M Gillis LA Yaeger D. Cornelia de Lange syndrome is caused by mutations in NIPBL, the human homolog of *Drosophila* melanogaster Nipped-B. Nat Genet. (2004) 36:631–5. 10.1038/ng1364 15146186 PMC4902017

[7] Pié J Puisac B Hernández-Marcos M Teresa-Rodrigo ME Gil-Rodríguez M Baquero-Montoya C. Special cases in Cornelia de Lange syndrome: the Spanish experience. Am J Med Genet C Semin Med Genet. (2016) 172:198–205. 10.1002/ajmg.c.31501 27164022

[8] Selicorni A Russo S Gervasini C Castronovo P Milani D Cavalleri F. Clinical score of 62 Italian patients with Cornelia de Lange syndrome and correlations with the presence and type of NIPBL mutation. Clin Genet. (2007) 72:98–108. 10.1111/j.1399-0004.2007.00832.x 17661813

[9] Ramos FJ Puisac B Baquero-Montoya C Gil-Rodríguez MC Bueno I Deardorff MA. Clinical utility gene card for: Cornelia de Lange syndrome. Eur J Hum Genet. (2015) 23:1431. 10.1038/ejhg.2014.270 25537356 PMC4592075

[10] Krawczynska N Kuzniacka A Wierzba J Parenti I Kaiser FJ Wasag B. Mosaic intronic NIPBL variant in a family with Cornelia de Lange syndrome. Front. Genet. (2018) 9:255. 10.3389/fgene.2018.00255 30057591 PMC6053508

[11] Teresa-Rodrigo ME Eckhold J Puisac B Pozojevic J Parenti I Baquero-Montoya C. Identification and functional characterization of two intronic NIPBL mutations in two patients with Cornelia de Lange syndrome. BioMed Res. Int. (2016) 2016:8742939. 10.1155/2016/8742939 26925417 PMC4746300

[12] Vaz-Drago R Custódio N Carmo-Fonseca M. Deep intronic mutations and human disease. Hum Genet. (2017) 136:1093–11. 10.1007/s00439-017-1809-4 28497172

[13] Richards S Aziz N Bale S Bick D Das S Gastier-Foster J. Standards and guidelines for the interpretation of sequence variants: a joint consensus recommendation of the American College of Medical Genetics and Genomics and the Association for Molecular Pathology. Genet Med. (2015) 17:405–24. 10.1038/gim.2015.30 25741868 PMC4544753

[14] Lewandowska MA The missing puzzle piece: splicing mutations. Int J Clin Exp Pathol. (2013) 15:2675–82. PMC3843248 24294354

[15] Ramanouskaya TV Grinev VV. The determinants of alternative RNA splicing in human cells. Mol Genet Genomics (2017) 292:1175–95. 10.1007/s00438-017-1350-0 28707092

[16] Borck G Zarhrate M Cluzeau C Bal E Bonnefont JP Munnich A. Father-to-daughter transmission of Cornelia de Lange syndrome caused by a mutation in the 5′ untranslated region of the NIPBL Gene. Hum Mut. (2006) 27:731–5. 10.1002/humu.20380 16799922

